# Prognostic and clinicopathological role of pretreatment systemic immune-inflammation index in patients with oral squamous cell carcinoma: a meta-analysis

**DOI:** 10.3389/fonc.2023.1303132

**Published:** 2024-01-16

**Authors:** Jiliang Zhang, Shu Dai

**Affiliations:** Clinical Laboratory, Lishui People’s Hospital, The Sixth Affiliated Hospital of Wenzhou Medical University, Lishui, Zhejiang, China

**Keywords:** SII, oral squamous cell carcinoma, meta-analysis, evidence-based medicine, prognostic markers

## Abstract

**Background:**

There are many studies regarding the use of systemic immune-inflammation index (SII) to help predict oral squamous cell carcinoma (OSCC) prognosis, but findings have been inconsistent. The present meta-analysis was conducted to determine whether SII could contribute to predicting OSCC prognosis.

**Methods:**

PubMed, Embase, Cochrane Library and Web of Science databases were thoroughly searched from their inceptions through August 20, 2023. The role of SII in predicting OSCC prognosis was determined through combined hazard ratios (HRs) with relevant 95% confidence intervals (CIs). Correlations of SII with clinicopathological characteristics of OSCC patients were analyzed based on combined odds ratios (ORs) with 95% CIs.

**Results:**

This meta-analysis utilized 11 articles in total, involving 3,464 patients. According to the results, an elevated SII was markedly associated with dismal overall survival (OS) (HR=1.85, 95%CI=1.48-2.29, p<0.001) and poor disease-free survival (DFS) (HR=1.77, 95%CI=1.20-2.61, p=0.004) of OSCC. Moreover, a higher SII was markedly correlated with stage T3-T4 (OR=2.47, 95%CI=1.40-4.37, p=0.002), TNM stage III-IV (OR=2.29, 95%CI=1.53-3.44, p<0.001), and low differentiation (OR=1.74, 95%CI=1.25-2.43, p=0.001).

**Conclusion:**

According to the present meta-analysis, an increased SII is significantly associated with dismal OS and DFS, advanced tumor stage and poor differentiation in OSCC. SII could be a potential and important biomarker for clinical management and predicting the prognosis of patients with OSCC.

**Systematic review registration:**

https://inplasy.com/inplasy-2023-9-0033/), identifier INPLASY202390033.

## Introduction

Head and neck cancer (HNC) is the sixth most common cancer across the world, affecting nearly 650,000 patients and contributing to 350,000 deaths every year (1, 2). Oral squamous cell carcinoma (OSCC), has the highest morbidity in HNC and constitutes 48% of all HNC cases (3). Moreover, OSCC includes cancers that occur in the lips, gums, tongue, mouth, and palate (4). Although there have been improvements in multidisciplinary collaboration and comprehensive therapy, such as surgery, radiotherapy, and chemotherapy, OSCC has had a low 5-year survival rate (under 50%) over the past two decades (5). Nowadays, the tumor-node-metastasis (TNM) classification system is widely used to guide the selection of treatment strategies and predict survival outcomes; however, patients of an identical TNM stage can have diverse disease courses (6). Therefore, identifying reliable and cost-effective prognostic markers for OSCC patients is urgently needed to intervene treatment measures and improve overall prognosis.

Accumulating evidence has shown that cancer-related immune and inflammatory responses have pivotal effects on tumor occurrence, growth, invasion, and progression (7). Many blood-based indexes that reflect inflammatory statuses have been identified as prognostic biomarkers in different cancer types. These indexes include neutrophil-to-lymphocyte ratio (NLR) (8), platelet-to-lymphocyte ratio (PLR) (9), C-reactive protein/albumin ratio (CAR) (10), lymphocyte-monocyte ratio (LMR) (11) and lymphocyte-to-C-reactive protein ratio (LCR) (12). Systemic immune-inflammation index (SII), a hematological parameter, is calculated by the following formula: SII = (platelet number × neutrophil number)/lymphocyte number. Moreover, SII has been widely demonstrated to significantly predict diverse cancer prognostic outcomes, such as thyroid cancer (13), cholangiocarcinoma (14), hepatocellular carcinoma (HCC) (15), glioma (16), and pancreatic cancer (17). The ability of SII to predict OSCC prognosis has been explored previously, but no consistent findings have been reported (18–28). For example, a higher SII was reported as a distinct prognostic indicator of OSCC in certain articles (19, 26, 28). In contrast, some researchers indicated the absence of any obvious association of SII with survival outcomes in OSCC (23–25). Consequently, to identify the precise impact of SII on predicting OSCC prognosis, this work carried out comprehensive literature retrieval for meta-analysis. Furthermore, the relationship between SII and clinicopathological features of OSCC patients was also investigated.

## Materials and methods

### Study guideline and protocol registration

The present study was carried out according to the Preferred Reporting Items for Systematic Reviews and Meta-Analyses (PRISMA) guideline (29), and registered in INPLASY (registration ID: INPLASY202390033, https://inplasy.com/inplasy-2023-9-0033/).

### Literature retrieval

Literature was retrieved from the PubMed, Embase, Cochrane Library and Web of Science databases, starting with the earliest possible date through August 20, 2023. The following terms were used to search and select literature for the meta-analysis: (systemic immune-inflammatory index or SII or systemic immune-inflammation index or systemic-immune-inflammation index) and (oral squamous cell carcinoma or OSCC or oral cancer or tongue cancer or mouth cancer or oral carcinoma or oral cavity cancer or lip cancer or gingiva cancer). More details about these search strategies are provided in **Supplementary File 1**. Only English publications were considered. Besides, references in each publication were manually retrieved to identify the possible relevant articles.

### Study eligibility criteria

Included studies had the following features (1): pathological diagnosis of primary OSCC (2); explored a relationship between pre-treatment SII and OSCC prognosis (3); hazard ratios (HRs) with 95% confidence intervals (CIs) can be determined according to the available data (4); the threshold SII is identified; and (5) articles written in the English language. Exclusion criteria were as follows (1): meeting abstracts, reviews, letters, comments, and case reports (2); does not have sufficient or available data (3); contains overlapped patients; and (4) animal studies.

### Data collection and quality evaluation

Qualified publications were evaluated by two independent reviewers (JZ, SD), who also extracted data. Any discrepancy was settled through negotiation until a consensus was reached. Data collected included, first author, publication year, study country/region, sample size, age, gender, study center, study design, study period, tumor subsite, TNM stage, treatment, threshold, threshold determination approach, survival outcomes, survival analysis type, follow-up, HRs and 95% CIs. Our primary and secondary outcomes were overall survival (OS) and disease-free survival (DFS), separately. We employed the Newcastle–Ottawa Scale (NOS) for assessing study quality (30). The NOS contains three perspectives, selection (0–4 points), comparability (0–2 points), and outcome assessment (0–3 points), with a total score of 0-9 points. NOS scores ≥ 6 indicate high-quality.

### Statistical analysis

Significance of SII in predicting OSCC prognosis was estimated based on combined HRs with 95% CIs. Additionally, I^2^ statistics and Cochrane’s Q test were utilized to evaluate inter-study heterogeneity. The random-effects model was utilized in the case of obvious heterogeneity (I^2^>50%, P<0.10), otherwise, a fixed-effects model was applied. The source of heterogeneity was detected by different factors-stratified subgroup analyses. Correlations of SII with clinicopathological characteristics of OSCC were evaluated through combined odds ratios (ORs) as well as 95% CIs. Sensitivity analysis was used to compare pooled effects, by eliminating one individual study in the sequence and observing any potential changes to the result, repeating the process for each study. We performed Egger’s and Begg’s tests for assessing publication bias, and conducted statistical analyses using Stata version 12.0 (Stata Corporation, College Station, TX, USA). P-values < 0.05 were defined as statistically significant differences.

## Results

### Study screening

**Figure 1:**
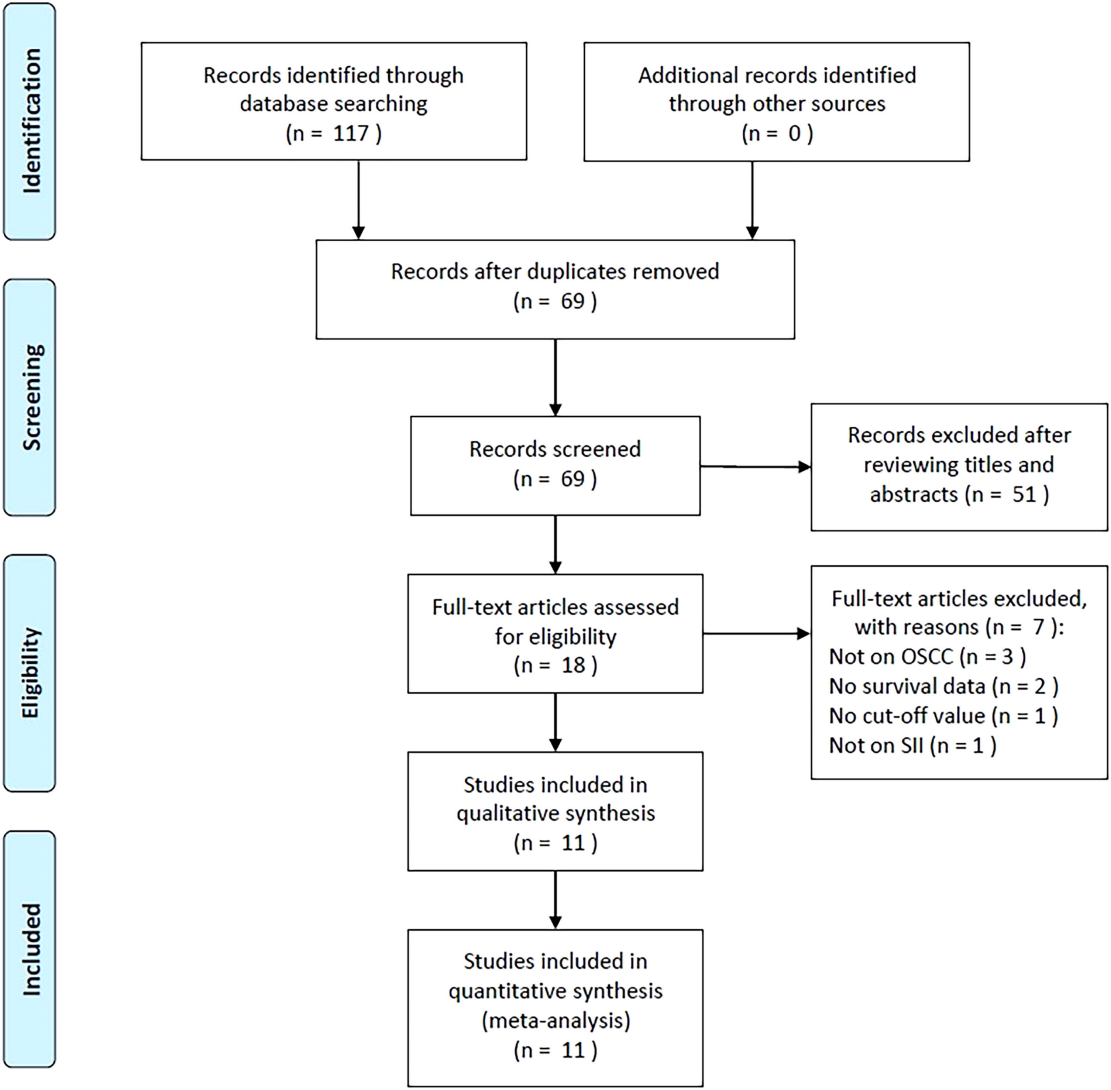
The PRISMA flowchart of study selection.

There were 117 articles obtained initially, among which 69 were retained following the removal of duplicates (**Figure 1**). Through title- and abstract-selection, 51 articles were then excluded due to irrelevance. Full-text review of the remaining 18 articles was conducted, among which, seven were eliminated for the following reasons, not focused on OSCC (n=3), no survival data provided (n=2), no cut-off value (n=1), and no report on SII (n=1). Ultimately, 11 articles were utilized for the remainder of the analysis, involving a total of 3,464 patients (18–28) (**Figure 1**, **Table 1**).

**Table 1:** The baseline characteristics of included studies in this meta-analysis.

Study	Year	Country/ region	Sample size	Age (years) Median(range)	Gender (M/F)	Study center	Study period	Tumor subsite	TNM stage	Treatment	Cut-off value	Cut-off determination	Survival endpoint	Survival analysis	Follow-up (month) Median(range)	NOS score
Diao, P.	2018	China	309	≤60 y: 112 >60 y: 197	171/138	Multicenter	2006-2016	Unspecified OSCC	I-IV	Surgery	484.5	X-tile	OS, DFS	Multivariate	48 (4–134)	9
Erdis, E.	2020	Turkey	58	67 (23–90)	40/18	Single center	2009-2018	Oral cavity	I-IV	RT	954	ROC curve	OS, DFS	Univariate	1-140	8
Lu, Z.	2020	China	120	55 (20–86)	79/41	Single center	2012-2017	Oral tongue	I-IV	Surgery	569	X-tile	OS, DFS	Multivariate	37.5(3-92)	8
Hung, S. P.	2021	Taiwan	993	51	922/71	Single center	2005-2012	Oral cavity	I-IV	Surgery+ RT/CCRT	810.6	ROC curve	OS	Multivariate	105.6	7
Nie, Z.	2021	China	269	62(21-85)	204/65	Single center	2007-2020	Unspecified OSCC	III-IV	Surgery	535.5	ROC curve	OS, DFS	Multivariate	55(2-95)	8
Wei, L. F.	2021	China	172	69(25-88)	96/76	Single center	2008-2019	Oral tongue	I-IV	Surgery	204	X-tile	OS	Univariate	65	7
Cho, U.	2022	Korea	269	55(18-90)	173/96	Single center	2003-2019	Unspecified OSCC	I-IV	Surgery	548.9	ROC curve	DFS	Multivariate	1-150	7
Huang, C. H.	2022	Taiwan	592	54	518/74	Single center	2011-2020	Unspecified OSCC	I-IV	Surgery	459	ROC curve	OS, DFS	Multivariate	100(6-173)	7
Kubota, K.	2022	Japan	183	66(26-93)	103/80	Single center	2005-2017	Unspecified OSCC	I-IV	Surgery+ RT/CCRT	569	Literature	OS, DFS	Univariate	1-150	8
Ruiz-Ranz, M.	2022	Spain	348	62(28-92)	221/127	Single center	1996-2007	Unspecified OSCC	I-IV	Surgery	1137	ROC curve	OS, DFS	Univariate	54(3-280)	7
Zakaria, S. S.	2022	Malaysia	151	59.7	56/95	Single center	2000-2020	Unspecified OSCC	I-IV	Surgery+ RT/CCRT	914	ROC curve	DFS	Multivariate	30(1-217)	8

M, male; F, female; OSCC, oral squamous cell carcinoma; OS, overall survival; DFS, disease-free survival; RT, radiotherapy; CCRT, concurrent chemoradiotherapy; ROC, receiver operating characteristic; NOS, Newcastle-Ottawa Scale.

### Enrolled study features

**Table 1** provides baseline study features (18–28). All included studies were retrospective in nature, published in the English language and had publication years ranging from 2018 to 2022. Four studies were carried out in China (18, 20, 22, 23), two in Taiwan (21, 25), and one each in Turkey (19), Korea (24), Japan (26), Spain (27), and Malaysia (28). Sample sizes ranged from 58-993 (median, 269). Ten articles described single center studies (19–28) and one was a multicenter study (18). Seven studies recruited patients with OSCC (18, 22, 24–28), two recruited oral cavity cancer cases (19, 21), and two involved tongue cancer cases (20, 23). Ten articles described studies involving patients with TNM stage I-IV (18–21, 23–28), whereas one study only included stage III-IV patients (22). Seven studies treated patients with surgery (18, 20, 22–25, 27), three studies used surgery and concurrent chemoradiotherapy (CCRT) (21, 26, 28), and one study only applied radiotherapy (RT) (19). The threshold SII ranged from 204-1,137 (median, 569) in all 11 studies. Seven articles described the threshold through receiver operating characteristic curve (19, 21, 22, 24, 25, 27, 28), three studies applied the X-tile software (18, 20, 23), whereas another one was determined using previous literature (26). Nine articles reported a prognostic effect of SII for OS (18–23, 25–27) and nine mentioned a relationship between SII and DFS (18–20, 22, 24–28) in OSCC. Seven articles mentioned HRs with 95% CIs based on multivariate regression (18, 20–22, 25, 26, 28) and four studies used univariate analyses (19, 23, 24, 27). For all enrolled articles, NOS scores were from 7-9 (median, 8), demonstrating high quality (**Table 1**).

### SII and OS of OSCC

**Figure 2:**
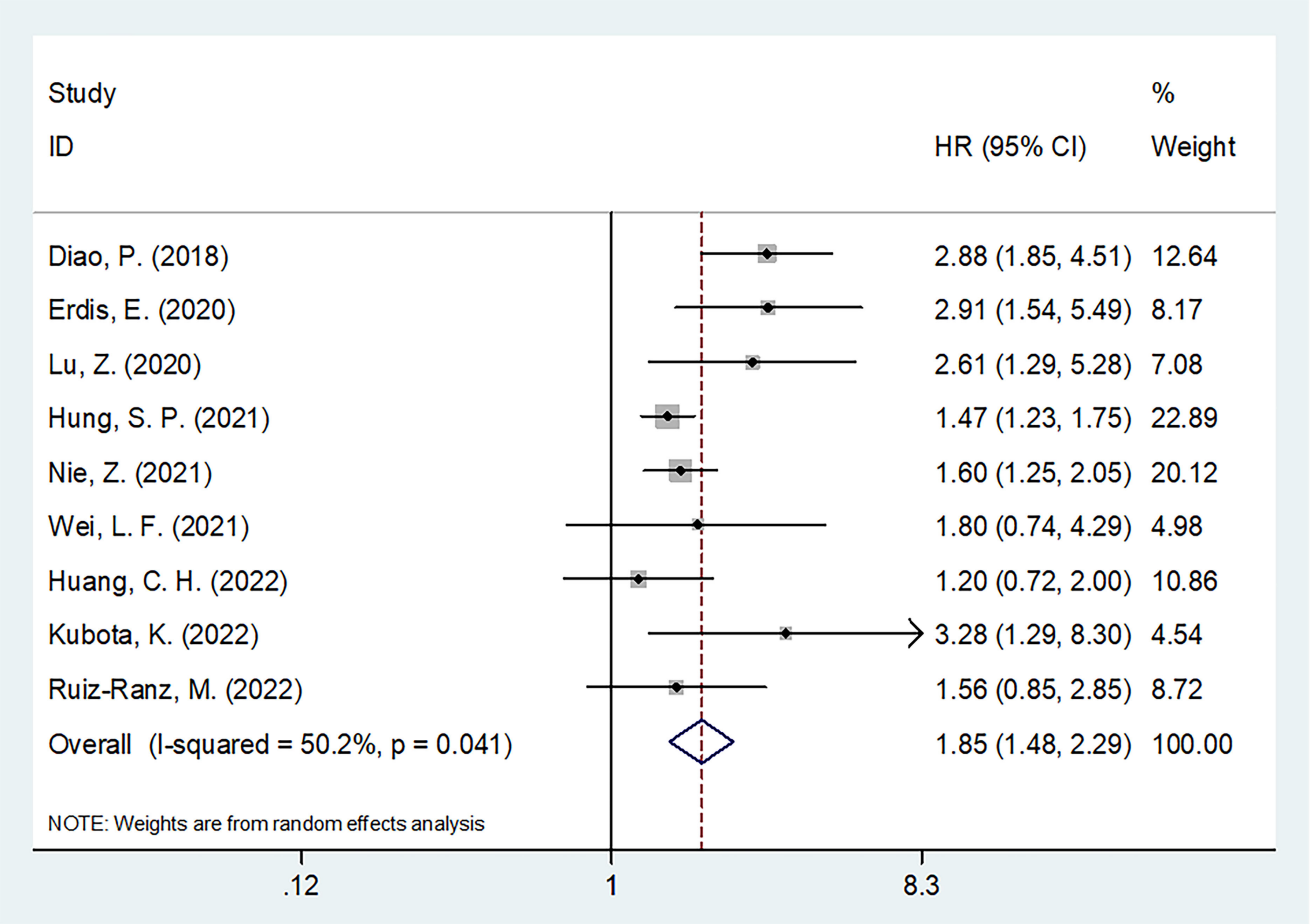
Forest plots on prognostic value of SII for overall survival in patients with OSCC.

Nine articles, involving 3,044 patients (18–23, 25–27), mentioned a significance of SII to predict OS in OSCC. Due to significant heterogeneity (I^2 =^ 50.2%, p=0.041), we selected the random-effects model. According to **Figure 2** and **Table 2**, HR=1.85, 95%CI=1.48-2.29, and p<0.001, which indicates that a higher SII was markedly related to the dismal OS of OSCC patients. According to subgroup analyses, sample size, study center, TNM stage, threshold, threshold determination method, and survival analysis type did not affect the significant role of SII to predict OS (p<0.05; **Table 2**). Moreover, higher SII still significantly predicted poor OS in the following subgroups: in Asian regions (p<0.001), tongue tumor site (p=0.004) or OSCC (p<0.001), and patients who received surgery (p<0.001) or RT (p=0.001) (**Table 2**).

**Table 2:** The subgroup analysis of the prognostic role of SII for OS in patients with OSCC.

Subgroups	No. of studies	No. of patients	Effects model	HR (95%CI)	p	Heterogeneity I^2^(%) Ph	
Total	9	3,044	Random	1.85(1.48-2.29)	<0.001	50.2	0.041
Geographical region							
Asian	8	2,696	Random	1.89(1.49-2.41)	<0.001	56.3	0.025
Non-Asian	1	348	–	1.56(0.85-2.85)	0.153	–	–
Sample size							
<300	5	802	Fixed	1.85(1.51-2.28)	<0.001	29.4	0.225
≥300	4	2,242	Random	1.67(1.18-2.37)	0.004	65.7	0.033
Study center							
Single center	8	2,735	Fixed	1.59(1.40-1.81)	<0.001	28.1	0.204
Multicenter	1	309	–	2.88(1.85-4.51)	<0.001	–	–
Tumor subsite							
Oral cavity	2	1,051	Random	1.92(1.00-3.71)	0.051	75.7	0.042
Oral tongue	2	292	Fixed	2.26(1.31-3.91)	0.004	0	0.516
Unspecified OSCC	5	1,701	Random	1.84(1.32-2.56)	<0.001	57.0	0.054
TNM stage							
I-IV	8	2,775	Random	1.96(1.48-2.60)	<0.001	56.1	0.026
III-IV	1	269	–	1.60(1.25-2.05)	<0.001	–	–
Treatment							
Surgery	6	1,810	Fixed	1.76(1.47-2.10)	<0.001	43.3	0.117
RT	1	58	–	2.91(1.54-5.49)	0.001	–	–
Surgery+ RT/CCRT	2	1,176	Random	1.92(0.91-4.03)	0.086	63.9	0.096
Cut-off value							
<569	4	1,374	Random	1.77(1.23-2.55)	0.002	59.4	0.060
≥569	5	1,670	Random	2.00(1.40-2.85)	<0.001	52.6	0.077
Cut-off selection							
ROC curve	5	2,260	Fixed	1.53(1.34-1.75)	<0.001	22.0	0.275
X-tile	3	601	Fixed	2.62(1.85-3.70)	<0.001	0	0.644
Literature	1	183	–	3.28(1.29-8.32)	0.012	–	–
Survival analysis							
Univariate	4	761	Fixed	2.19(1.52-3.14)	<0.001	0	0.408
Multivariate	5	2,283	Random	1.73(1.34-2.24)	<0.001	62.6	0.030

SII, systemic immune-inflammation index; OS, overall survival; OSCC, oral squamous cell carcinoma; ROC, receiver operating characteristic; RT, radiotherapy; CCRT, concurrent chemoradiotherapy.

### SII and DFS in OSCC

**Figure 3:**
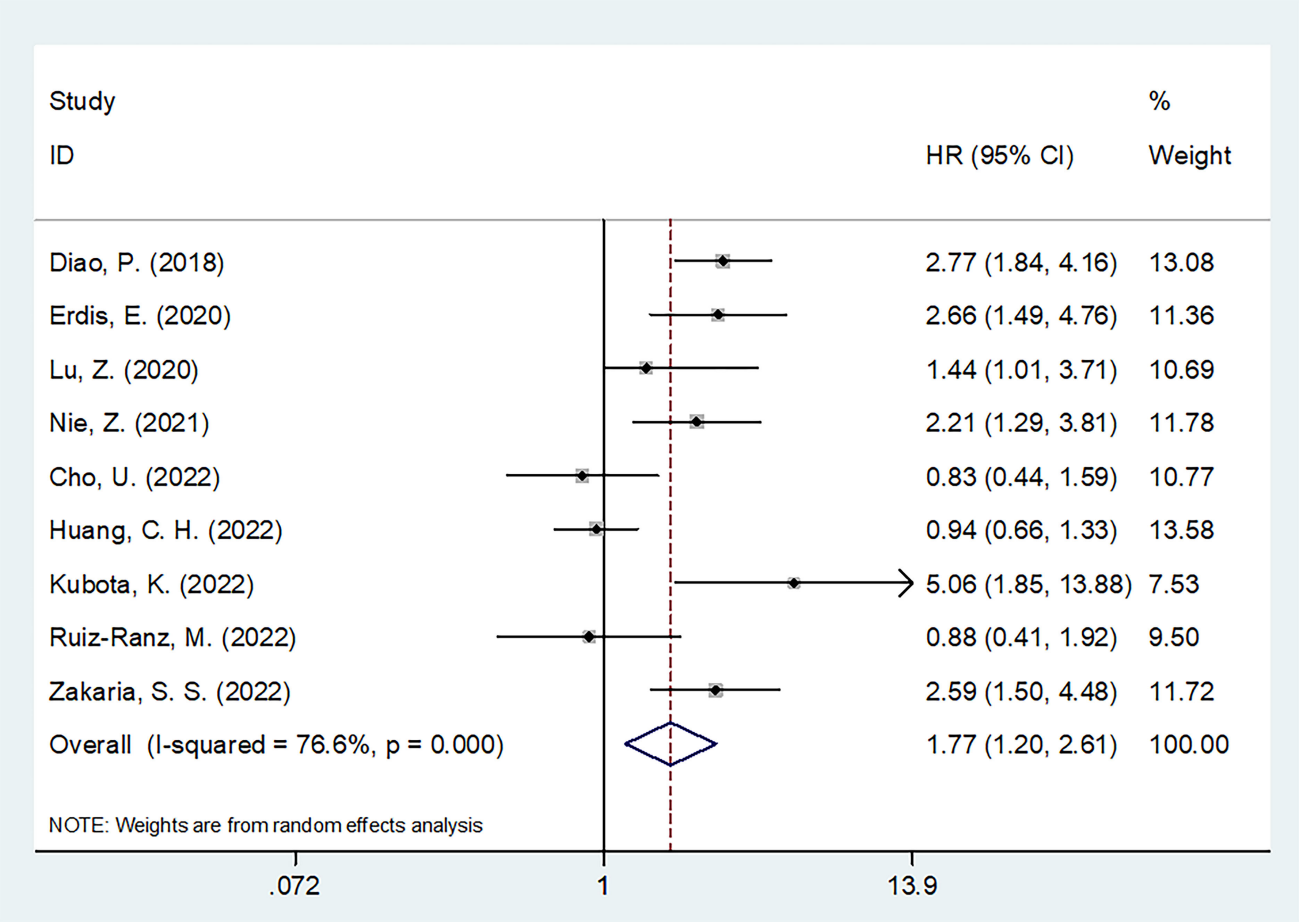
Forest plots on prognostic value of SII for disease-free survival in patients with OSCC.

Altogether, nine articles, involving 2,299 patients (18–20, 22, 24–28), mentioned the prognostic effect of SII for DFS in OSCC. Based on our pooled results, higher SII was significantly related to inferior DFS in OSCC (HR=1.77, 95%CI=1.20-2.61, p=0.004) (**Figure 3**; **Table 3**). According to subgroup analyses, high SII significantly predicted DFS, and remained unaffected by the study center or TNM stage (p<0.05; **Table 3**). Additionally, elevated SII was markedly related to dismal DFS for the following subgroups: in Asian regions (p=0.002), sample size < 300 (p=0.001), multicenter studies (p<0.001), oral cavity tumor site (p=0.001) or OSCC (p=0.026), patients who received RT (p=0.001) or surgery + CCRT (p<0.001), SII threshold ≥ 569 (p=0.004), threshold determined by X-tile (p=0.022) or literature (p=0.002), and multivariate analysis (p=0.034) (**Table 3**).

**Table 3:** The subgroup analysis of the prognostic role of SII for DFS in patients with OSCC.

Subgroups	No. of studies	No. of patients	Effects model	HR (95%CI)	p	Heterogeneity I^2^(%) Ph	
Total	9	2,299	Random	1.77(1.20-2.61)	0.004	76.6	<0.001
Geographical region							
Asian	8	1,951	Random	1.90(1.26-2.86)	0.002	77.7	<0.001
Non-Asian	1	348	–	0.88(0.41-1.92)	0.753	–	–
Sample size							
<300	6	1,050	Random	2.03(1.33-3.11)	0.001	62.6	0.020
≥300	3	1,249	Random	1.35(0.61-3.01)	0.459	88.3	<0.001
Study center							
Single center	8	1,990	Random	1.65(1.09-2.50)	0.017	74.1	<0.001
Multicenter	1	309	–	2.77(1.84-4.16)	<0.001	–	–
Tumor subsite							
Oral cavity	1	58	–	2.66(1.49-4.76)	0.001	–	–
Oral tongue	1	120	–	1.44(0.75-2.76)	0.273	–	–
Unspecified OSCC	7	2,121	Random	1.72(1.07-2.77)	0.026	80.9	<0.001
TNM stage							
I-IV	8	2,030	Random	1.72(1.11-2.66)	0.015	78.8	<0.001
III-IV	1	269	–	2.21(1.29-3.80)	0.004	–	–
Treatment							
Surgery	6	1,907	Random	1.38(0.88-2.18)	0.161	77.6	<0.001
RT	1	58	–	2.66(1.49-4.76)	0.001	–	–
Surgery+ RT/CCRT	2	334	Fixed	3.02(1.87-4.88)	<0.001	23.4	0.253
Cut-off value							
<569	4	1,439	Random	1.49(0.81-2.76)	0.201	85.6	<0.001
≥569	5	860	Random	2.07(1.27-3.39)	0.004	60.9	0.037
Cut-off selection							
ROC curve	6	1,687	Random	1.49(0.94-2.37)	0.087	76.4	0.001
X-tile	2	429	Random	2.10(1.12-3.95)	0.022	62.4	0.095
Literature	1	183	–	5.06(1.85-13.86)	0.002	–	–
Survival analysis							
Univariate	3	589	Random	2.21(0.89-5.51)	0.089	76.1	0.015
Multivariate	6	1,710	Random	1.63(1.04-2.56)	0.034	79.4	<0.001

SII, systemic immune-inflammation index; DFS, disease-free survival; OSCC, oral squamous cell carcinoma; ROC, receiver operating characteristic; RT, radiotherapy; CCRT, concurrent chemoradiotherapy.

### Association of SII with clinicopathological characteristics of OSCC

**Figure 4:**
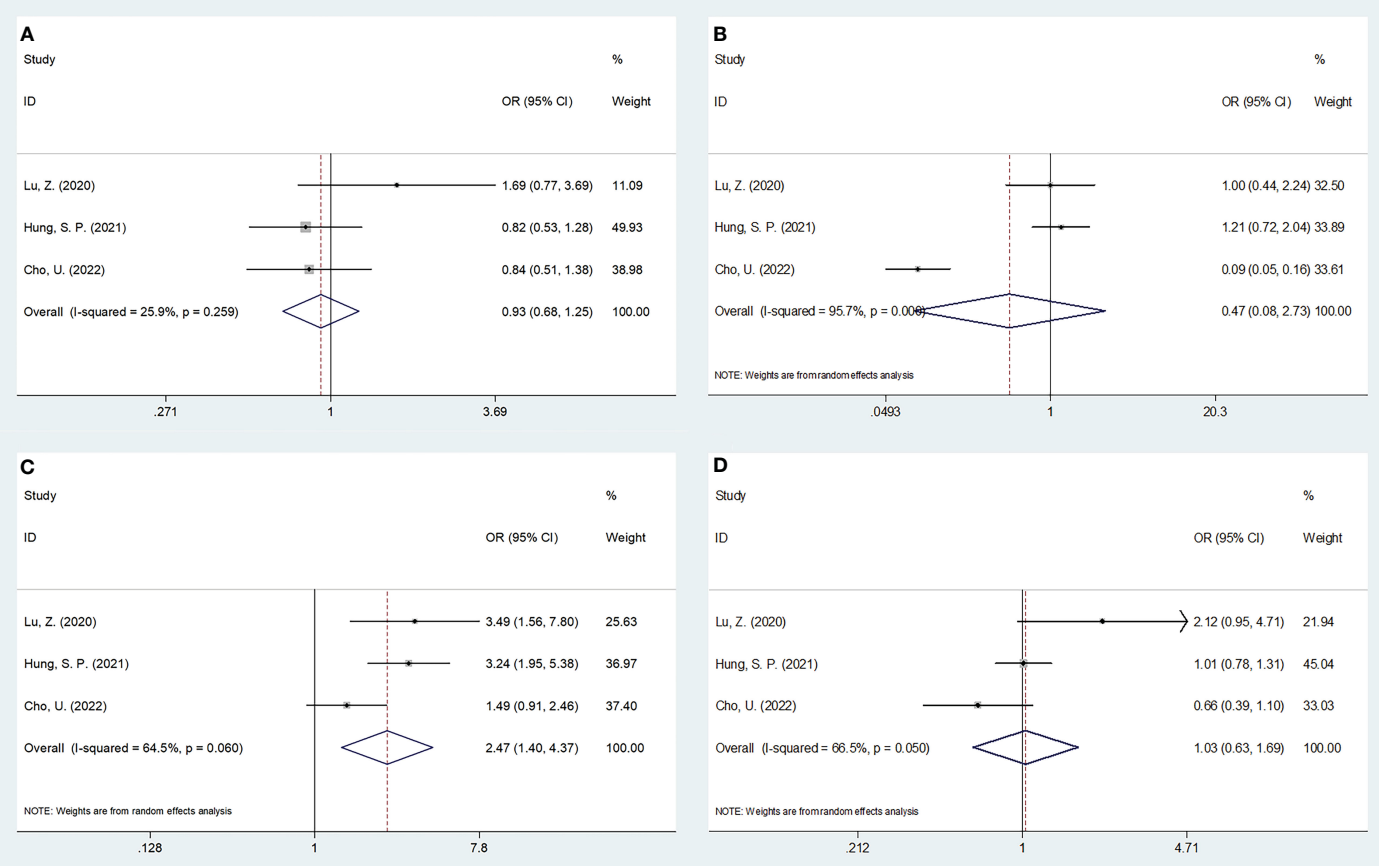
Forest plots on the association between SII and clinicopathological features in OSCC. **(A)** age (year) (≥55 vs <55); **(B)** gender (male vs female); **(C)** T stage (T3-T4 vs T1-T2); and **(D)** lymph node metastasis (yes vs no).

**Figure 5:**
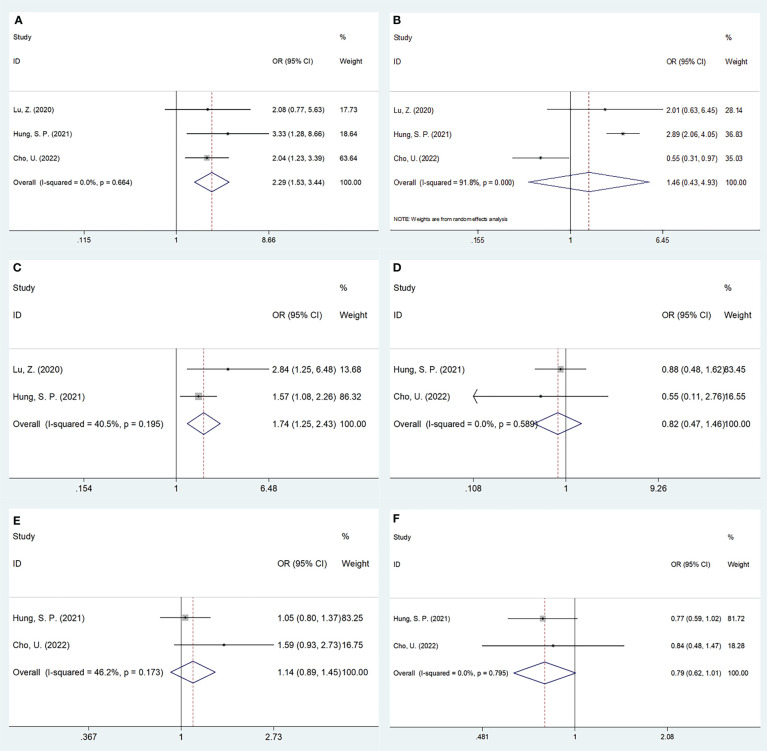
Forest plots on the association between SII and clinicopathological features in OSCC. **(A)** TNM stage (III-IV vs I-II); **(B)** depth of invasion (>1cm vs ≤1cm); **(C)** tumor differentiation (poor vs well/moderate); **(D)** vascular invasion (yes vs no); **(E)** perineural invasion (yes vs no); and **(F)** tumor site (tongue vs others).

Three studies, encompassing 1,382 patients (20, 21, 24), presented data explaining a relationship of SII with OSCC clinicopathological features. According to the combined results, shown in **Table 4**, **Figures 4** and **5**, higher SII was remarkably related to stages T3-T4 (OR=2.47, 95%CI=1.40-4.37, p=0.002), TNM stages III-IV (OR=2.29, 95%CI=1.53-3.44, p<0.001), and low differentiation (OR=1.74, 95%CI=1.25-2.43, p=0.001). However, SII did not show any significant correlation with age (OR=0.93, 95%CI=0.68-1.25, p=0.617), gender (OR=0.47, 95%CI=0.08-2.73, p=0.402), tumor site (OR=0.79, 95%CI=0.62-1.01, p=0.056), lymph node metastasis (OR=1.03, 95%CI=0.63-1.69, p=0.906), invasion depth (OR=1.46, 95%CI=0.43-4.93, p=0.545), vascular invasion (OR=0.82, 95%CI=0.47-1.46, p=0.506), or perineural invasion (OR=1.14, 95%CI=0.89-1.45, p=0.297) (**Table 4**, **Figures 4**, **5**).

**Table 4:** The association between SII and clinicopathological features in patients with OSCC.

Variables	No. of studies	No. of patients	Effects model	OR (95%CI)	p	Heterogeneity I^2^(%) Ph	
Age (year) (≥55 vs <55)	3	1,382	Fixed	0.93(0.68-1.25)	0.617	25.9	0.259
Gender (male vs female)	3	1,382	Random	0.47(0.08-2.73)	0.402	95.7	<0.001
T stage (T3-T4 vs T1-T2)	3	1,382	Random	2.47(1.40-4.37)	0.002	64.5	0.060
LN metastasis (yes vs no)	3	1,382	Random	1.03(0.63-1.69)	0.906	66.5	0.050
TNM stage (III-IV vs I-II)	3	1,382	Fixed	2.29(1.53-3.44)	<0.001	0	0.664
Depth of invasion (>1cm vs ≤1cm)	3	1,382	Random	1.46(0.43-4.93)	0.545	91.8	<0.001
Tumor differentiation (poor vs well/moderate)	2	1,113	Fixed	1.74(1.25-2.43)	0.001	40.5	0.195
Vascular invasion (yes vs no)	2	1,262	Fixed	0.82(0.47-1.46)	0.506	0	0.589
Perineural invasion (yes vs no)	2	1,262	Fixed	1.14(0.89-1.45)	0.297	46.2	0.173
Tumor site (tongue vs others)	2	1,262	Fixed	0.79(0.62-1.01)	0.056	0	0.795

SII, systemic immune-inflammation index; OS, overall survival; OSCC, oral squamous cell carcinoma; LN, lymph node; TNM, tumor (T), node (N), and metastasis (M).

### Sensitivity analysis

**Figure 6:**
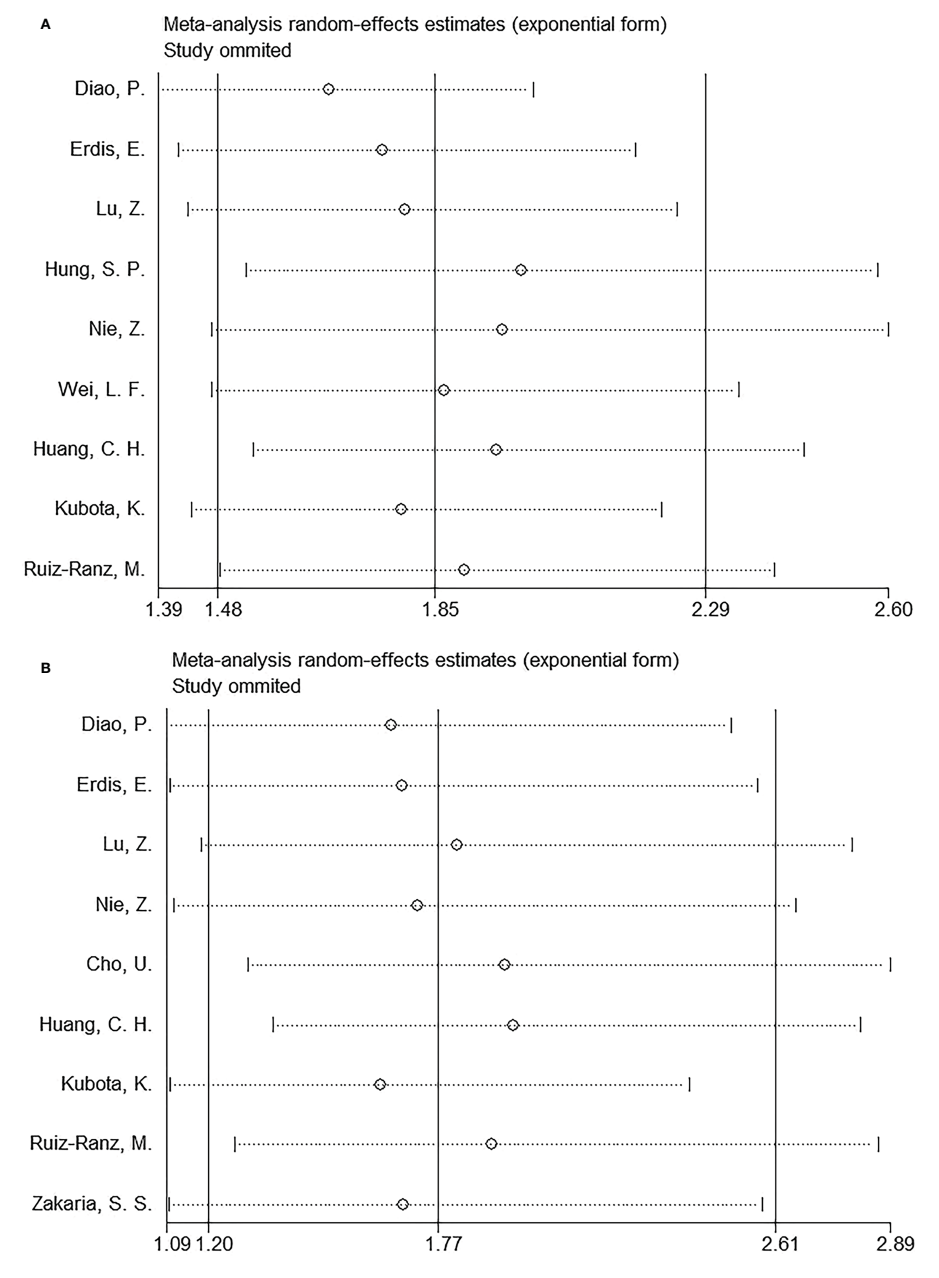
Sensitivity analysis. **(A)** OS; and **(B)** DFS.

Every article was removed individually during each sensitivity analysis. Results were recalculated each time, based on the remaining studies’ OS and DFS. According to **Figure 6**, in the overall analysis of OS and DFS, there was no significant difference after eliminating each work, suggesting the reliability of our combined results.

### Publication bias

**Figure 7:**
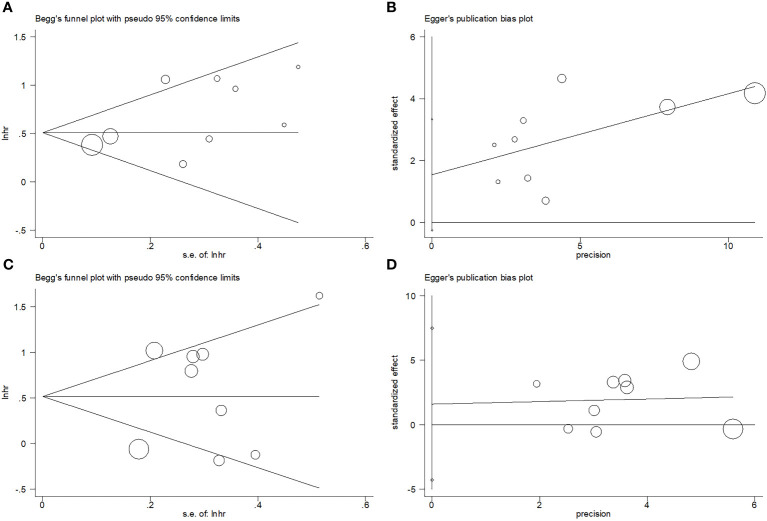
Publication bias test. **(A)** Begg’s test for OS, p=0.175; **(B)** Egger’s test for OS, p=0.082; **(C)** Begg’s test for DFS, p=1; and **(D)** Egger’s test for DFS, p=0.542.

Begg’s funnel plots and the Egger’s test were conducted to assess possible publication bias. The funnel plots observed in **Figure 7** show symmetry, suggesting no significant publication bias for OS (p=0.175 and p=0.082 upon Begg’s and Egger’s tests, separately) or DFS (p=1 and p=0.542 upon Begg’s and Egger’s tests, separately).

## Discussion

Previously, the effect of SII to predict OSCC prognosis has been explored, but no consistent findings have been reported (18–28). This work combined results from 11 articles involving 3,464 patients. According to our results, an elevated SII was remarkably related to dismal OS and inferior DFS of OSCC. Moreover, SII had a stable role when predicting prognosis, as examined by sensitivity, subgroup, and publication basis analyses. Higher SII was also evidently related to T3-T4, TNM III-IV, and poor tumor differentiation. Taken together, a higher SII significantly predicted the short- and long-term survival of OSCC, which was also dramatically related to tumor metastasis and poor differentiation. To our knowledge, this is the first meta-analysis investigating whether SII could be used to predict OSCC prognosis.

To understand the biological mechanism behind SII’s prognostic value, it is necessary to understand the function of neutrophils, platelets, and lymphocytes. First, neutrophils release inflammatory mediators such as neutrophil elastase, interleukin-8 (IL-8) and matrix metalloproteinase-9 (MMP-9) which enhance tumor cell growth, migration and invasion (31). Increased neutrophil counts can also produce reactive oxygen species, nitric oxide, and arginase, resulting in disordered T cell activation (32). Consequently, the body loses its ability to target tumor cells, indirectly contributing to tumor progression (33). Second, platelets can protect cancer cells from natural killer cells and tumor necrosis factor-α (TNF-α) by using glycoprotein (GP) receptors and tumor cell integrin α vβ-dependent pathway (34). Platelets also induce epithelial-mesenchymal transition and support transendothelial migration in circulating tumor cells, ultimately protecting tumor cells from immune destruction and promoting distant metastasis (35, 36). Third, lymphocytes are responsible for the adaptive immune response and participate in cancer immunosurveillance and immunoediting. Tumor-infiltrating lymphocytes promote tumor cell apoptosis and remove dead cells by way of humoral and cellular immunity, and these processes are necessary for the host’s immune defense and surveillance (37). Therefore, SII has a biological rationale for its role in predicting OSCC prognosis. Notably, a recent single study by Yoshimura et al. investigated the prognostic effect of multiple inflammation-nutrition parameters including NLR, PLR, LMR, CRP-albumin ratio (CAR), Glasgow prognostic score (GPS), modified GPS (mGPS), prognostic nutritional index (PNI), controlling nutrition status (CONUT), and modified CONUT (mCONUT) in patients with OSCC receiving surgery (38). They found that a low PNI was associated with shorter OS and DFS in patients with OSCC through multivariate analysis (38). Although that study did not include SII for analysis in OSCC, their results were important to investigate mechanisms (38). In peripheral blood analyses, inflammation-related markers were mainly composed of upregulated factors (neutrophils, platelets, monocytes, and CRP) and downregulated factors (lymphocytes, albumin, total cholesterol, and hemoglobin). Different combinations of these factors became prognostic indicators and the prognostic parameters were more stable than using a single element.

Many recent studies have also reported that SII could be used to predict the prognosis of different cancer types by conducting meta-analyses (39–43). A meta-analysis on 2,101 patients conducted by Zeng et al. found that elevated pretreatment SII was markedly associated with poor OS and progression-free survival (PFS) in esophageal squamous cell carcinoma (39). According to Wang et al., SII could independently predict OS and PFS of nasopharyngeal carcinoma patients through a meta-analysis that included nine studies (40). In the meta-analysis, which included 833 patients conducted by Salazar-Valdivia et al., indicated that high SII values are related to poor OS and PFS of testicular cancer (41). Moreover, a recent meta-analysis, including 1,426 patients, indicated that higher SII was significantly related to dismal OS and PFS in glioma patients (42). According to Zhang et al., a higher SII is linked dramatically to dismal OS and worse PFS/biochemical recurrence-free survival (bRFS) of prostate cancer in their meta-analysis enrolling 8,133 patients (43). The results of this SII focused meta-analysis mostly conforms to those obtained in additional cancer types.

There were some limitations to be noted. First, every enrolled article had a retrospective design, which could introduce selection bias. Second, many enrolled articles were conducted in Asia (10 out of 11). Although the study region was not restricted, all included studies were published in English. Therefore, the findings of this work may be more applicable in Asian OSCC populations. Third, threshold SII was not uniform across the included studies, so there could be differences to each conclusion. Due to these limitations, more multi-regional prospective trials are still necessary to further validate the utility of SII when predicting the prognosis of OSCC patients.

## Conclusions

In conclusion, this meta-analysis demonstrates that higher SIIs are significantly related to dismal OS and DFS in OSCC. Additionally, high SIIs are markedly related to advanced tumor stages and poor differentiation in OSCC. SII could be a potential and important biomarker for clinical management and prognosis prediction of OSCC patients.

## Data availability statement

The original contributions presented in the study are included in the article/**Supplementary Material**. Further inquiries can be directed to the corresponding author.

## Author contributions

JZ: Conceptualization, Data curation, Formal analysis, Investigation, Methodology, Project administration, Resources, Software, Supervision, Validation, Visualization, Writing – original draft. SD: Conceptualization, Investigation, Methodology, Project administration, Resources, Software, Validation, Visualization, Writing – review & editing.
