# Supplementary material for: Prognostic and clinicopathological role of pretreatment systemic immune-inflammation index in patients with oral squamous cell carcinoma: a meta-analysis
Source: Front Oncol. 2024 Jan 16;13:1303132. doi: 10.3389/fonc.2023.1303132 (PMC10824905; doi:10.3389/fonc.2023.1303132)
Supplement: Supplementary file 1 [file DataSheet_1.docx]

**Supplemental file 1. The detailed search strategies for each database.**

**1. Search strategies for PubMed:**

Search: (systemic immune-inflammatory index or SII or systemic-immune-inflammation index or systemic immune-inflammation index) and (oral squamous cell carcinoma or OSCC or tongue cancer or oral cancer or oral carcinoma or mouth cancer or oral cavity cancer or gingiva cancer or lip cancer) Sort by: Most Recent

((("systemic"[All Fields] OR "systemically"[All Fields] OR "systemics"[All Fields]) AND "immune-inflammatory"[All Fields] AND ("abstracting and indexing"[MeSH Terms] OR ("abstracting"[All Fields] AND "indexing"[All Fields]) OR "abstracting and indexing"[All Fields] OR "index"[All Fields] OR "indexed"[All Fields] OR "indexes"[All Fields] OR "indexing"[All Fields] OR "indexation"[All Fields] OR "indexations"[All Fields] OR "indexe"[All Fields] OR "indexer"[All Fields] OR "indexers"[All Fields] OR "indexs"[All Fields])) OR ("stat interface"[Journal] OR "sii"[All Fields]) OR ("systemic-immune-inflammation"[All Fields] AND ("abstracting and indexing"[MeSH Terms] OR ("abstracting"[All Fields] AND "indexing"[All Fields]) OR "abstracting and indexing"[All Fields] OR "index"[All Fields] OR "indexed"[All Fields] OR "indexes"[All Fields] OR "indexing"[All Fields] OR "indexation"[All Fields] OR "indexations"[All Fields] OR "indexe"[All Fields] OR "indexer"[All Fields] OR "indexers"[All Fields] OR "indexs"[All Fields])) OR (("systemic"[All Fields] OR "systemically"[All Fields] OR "systemics"[All Fields]) AND "immune-inflammation"[All Fields] AND ("abstracting and indexing"[MeSH Terms] OR ("abstracting"[All Fields] AND "indexing"[All Fields]) OR "abstracting and indexing"[All Fields] OR "index"[All Fields] OR "indexed"[All Fields] OR "indexes"[All Fields] OR "indexing"[All Fields] OR "indexation"[All Fields] OR "indexations"[All Fields] OR "indexe"[All Fields] OR "indexer"[All Fields] OR "indexers"[All Fields] OR "indexs"[All Fields]))) AND ("squamous cell carcinoma of head and neck"[MeSH Terms] OR ("squamous"[All Fields] AND "cell"[All Fields] AND "carcinoma"[All Fields] AND "head"[All Fields] AND "neck"[All Fields]) OR "squamous cell carcinoma of head and neck"[All Fields] OR ("oral"[All Fields] AND "squamous"[All Fields] AND "cell"[All Fields] AND "carcinoma"[All Fields]) OR "oral squamous cell carcinoma"[All Fields] OR "OSCC"[All Fields] OR ("tongue neoplasms"[MeSH Terms] OR ("tongue"[All Fields] AND "neoplasms"[All Fields]) OR "tongue neoplasms"[All Fields] OR ("tongue"[All Fields] AND "cancer"[All Fields]) OR "tongue cancer"[All Fields]) OR ("mouth neoplasms"[MeSH Terms] OR ("mouth"[All Fields] AND "neoplasms"[All Fields]) OR "mouth neoplasms"[All Fields] OR ("oral"[All Fields] AND "cancer"[All Fields]) OR "oral cancer"[All Fields]) OR (("mouth"[MeSH Terms] OR "mouth"[All Fields] OR "oral"[All Fields]) AND ("carcinoma"[MeSH Terms] OR "carcinoma"[All Fields] OR "carcinomas"[All Fields] OR "carcinoma s"[All Fields])) OR ("mouth neoplasms"[MeSH Terms] OR ("mouth"[All Fields] AND "neoplasms"[All Fields]) OR "mouth neoplasms"[All Fields] OR ("mouth"[All Fields] AND "cancer"[All Fields]) OR "mouth cancer"[All Fields]) OR ("mouth neoplasms"[MeSH Terms] OR ("mouth"[All Fields] AND "neoplasms"[All Fields]) OR "mouth neoplasms"[All Fields] OR ("oral"[All Fields] AND "cavity"[All Fields] AND "cancer"[All Fields]) OR "oral cavity cancer"[All Fields]) OR (("gingiva"[MeSH Terms] OR "gingiva"[All Fields] OR "gingivae"[All Fields]) AND ("cancer s"[All Fields] OR "cancerated"[All Fields] OR "canceration"[All Fields] OR "cancerization"[All Fields] OR "cancerized"[All Fields] OR "cancerous"[All Fields] OR "neoplasms"[MeSH Terms] OR "neoplasms"[All Fields] OR "cancer"[All Fields] OR "cancers"[All Fields])) OR ("lip neoplasms"[MeSH Terms] OR ("lip"[All Fields] AND "neoplasms"[All Fields]) OR "lip neoplasms"[All Fields] OR ("lip"[All Fields] AND "cancer"[All Fields]) OR "lip cancer"[All Fields]))

Translations

systemic: "systemic"[All Fields] OR "systemically"[All Fields] OR "systemics"[All Fields]

index: "abstracting and indexing"[MeSH Terms] OR ("abstracting"[All Fields] AND "indexing"[All Fields]) OR "abstracting and indexing"[All Fields] OR "index"[All Fields] OR "indexed"[All Fields] OR "indexes"[All Fields] OR "indexing"[All Fields] OR "indexation"[All Fields] OR "indexations"[All Fields] OR "indexe"[All Fields] OR "indexer"[All Fields] OR "indexers"[All Fields] OR "indexs"[All Fields]

SII: "Stat Interface"[Journal:__jid101471232] OR "sii"[All Fields]

index: "abstracting and indexing"[MeSH Terms] OR ("abstracting"[All Fields] AND "indexing"[All Fields]) OR "abstracting and indexing"[All Fields] OR "index"[All Fields] OR "indexed"[All Fields] OR "indexes"[All Fields] OR "indexing"[All Fields] OR "indexation"[All Fields] OR "indexations"[All Fields] OR "indexe"[All Fields] OR "indexer"[All Fields] OR "indexers"[All Fields] OR "indexs"[All Fields]

systemic: "systemic"[All Fields] OR "systemically"[All Fields] OR "systemics"[All Fields]

index: "abstracting and indexing"[MeSH Terms] OR ("abstracting"[All Fields] AND "indexing"[All Fields]) OR "abstracting and indexing"[All Fields] OR "index"[All Fields] OR "indexed"[All Fields] OR "indexes"[All Fields] OR "indexing"[All Fields] OR "indexation"[All Fields] OR "indexations"[All Fields] OR "indexe"[All Fields] OR "indexer"[All Fields] OR "indexers"[All Fields] OR "indexs"[All Fields]

oral squamous cell carcinoma: "squamous cell carcinoma of head and neck"[MeSH Terms] OR ("squamous"[All Fields] AND "cell"[All Fields] AND "carcinoma"[All Fields] AND "head"[All Fields] AND "neck"[All Fields]) OR "squamous cell carcinoma of head and neck"[All Fields] OR ("oral"[All Fields] AND "squamous"[All Fields] AND "cell"[All Fields] AND "carcinoma"[All Fields]) OR "oral squamous cell carcinoma"[All Fields]

tongue cancer: "tongue neoplasms"[MeSH Terms] OR ("tongue"[All Fields] AND "neoplasms"[All Fields]) OR "tongue neoplasms"[All Fields] OR ("tongue"[All Fields] AND "cancer"[All Fields]) OR "tongue cancer"[All Fields]

oral cancer: "mouth neoplasms"[MeSH Terms] OR ("mouth"[All Fields] AND "neoplasms"[All Fields]) OR "mouth neoplasms"[All Fields] OR ("oral"[All Fields] AND "cancer"[All Fields]) OR "oral cancer"[All Fields]

oral: "mouth"[MeSH Terms] OR "mouth"[All Fields] OR "oral"[All Fields]

carcinoma: "carcinoma"[MeSH Terms] OR "carcinoma"[All Fields] OR "carcinomas"[All Fields] OR "carcinoma's"[All Fields]

mouth cancer: "mouth neoplasms"[MeSH Terms] OR ("mouth"[All Fields] AND "neoplasms"[All Fields]) OR "mouth neoplasms"[All Fields] OR ("mouth"[All Fields] AND "cancer"[All Fields]) OR "mouth cancer"[All Fields]

oral cavity cancer: "mouth neoplasms"[MeSH Terms] OR ("mouth"[All Fields] AND "neoplasms"[All Fields]) OR "mouth neoplasms"[All Fields] OR ("oral"[All Fields] AND "cavity"[All Fields] AND "cancer"[All Fields]) OR "oral cavity cancer"[All Fields]

gingiva: "gingiva"[MeSH Terms] OR "gingiva"[All Fields] OR "gingivae"[All Fields]

cancer: "cancer's"[All Fields] OR "cancerated"[All Fields] OR "canceration"[All Fields] OR "cancerization"[All Fields] OR "cancerized"[All Fields] OR "cancerous"[All Fields] OR "neoplasms"[MeSH Terms] OR "neoplasms"[All Fields] OR "cancer"[All Fields] OR "cancers"[All Fields]

lip cancer: "lip neoplasms"[MeSH Terms] OR ("lip"[All Fields] AND "neoplasms"[All Fields]) OR "lip neoplasms"[All Fields] OR ("lip"[All Fields] AND "cancer"[All Fields]) OR "lip cancer"[All Fields]

**2. Search strategies for Web of Science:**

(systemic immune-inflammatory index or SII or systemic-immune-inflammation index or systemic immune-inflammation index) and (oral squamous cell carcinoma or OSCC or tongue cancer or oral cancer or oral carcinoma or mouth cancer or oral cavity cancer or gingiva cancer or lip cancer) (all fields)

Search link：https://www.webofscience.com/wos/woscc/summary/64be0aa5-c276-469d-b85f-1d984a0d54ae-a2acab51/recently-added/1

**3. Search strategies for Embase:**

('systemic immune-inflammatory index' OR (systemic AND 'immune inflammatory' AND ('index'/exp OR index)) OR sii OR 'systemic-immune-inflammation index' OR (('systemic immune inflammation'/exp OR 'systemic immune inflammation') AND ('index'/exp OR index)) OR 'systemic immune-inflammation index' OR (systemic AND 'immune inflammation' AND ('index'/exp OR index))) AND ('oral squamous cell carcinoma'/exp OR 'oral squamous cell carcinoma' OR (oral AND squamous AND ('cell'/exp OR cell) AND ('carcinoma'/exp OR carcinoma)) OR oscc OR 'tongue cancer'/exp OR 'tongue cancer' OR (('tongue'/exp OR tongue) AND ('cancer'/exp OR cancer)) OR 'oral cancer'/exp OR 'oral cancer' OR (oral AND ('cancer'/exp OR cancer)) OR 'oral carcinoma'/exp OR 'oral carcinoma' OR (oral AND ('carcinoma'/exp OR carcinoma)) OR 'mouth cancer'/exp OR 'mouth cancer' OR (('mouth'/exp OR mouth) AND ('cancer'/exp OR cancer)) OR 'oral cavity cancer'/exp OR 'oral cavity cancer' OR (oral AND cavity AND ('cancer'/exp OR cancer)) OR 'gingiva cancer'/exp OR 'gingiva cancer' OR (('gingiva'/exp OR gingiva) AND ('cancer'/exp OR cancer)) OR 'lip cancer'/exp OR 'lip cancer' OR (('lip'/exp OR lip) AND ('cancer'/exp OR cancer)))

**4.** **Search strategies for Cochrane Library:**

(systemic immune-inflammatory index or SII or systemic-immune-inflammation index or systemic immune-inflammation index) and (oral squamous cell carcinoma or OSCC or tongue cancer or oral cancer or oral carcinoma or mouth cancer or oral cavity cancer or gingiva cancer or lip cancer)in Title Abstract Keyword
